# Carbon nanomaterials sensitize prostate cancer cells to docetaxel and mitomycin C via induction of apoptosis and inhibition of proliferation

**DOI:** 10.3762/bjnano.8.132

**Published:** 2017-06-23

**Authors:** Kati Erdmann, Jessica Ringel, Silke Hampel, Manfred P Wirth, Susanne Fuessel

**Affiliations:** 1 Department of Urology, Medizinische Fakultät Carl Gustav Carus, Technische Universität Dresden, Fetscherstraße 74, Dresden 01307, Germany; 2 Leibniz Institute of Solid State and Material Research Dresden, P.O. Box 270016, Dresden 01171, Germany

**Keywords:** carbon nanomaterials, chemosensitization, docetaxel, mitomycin C, prostate cancer cells

## Abstract

We have previously shown that carbon nanofibers (CNFs) and carbon nanotubes (CNTs) can sensitize prostate cancer (PCa) cells to platinum-based chemotherapeutics. In order to further verify this concept and to avoid a bias, the present study investigates the chemosensitizing potential of CNFs and CNTs to the conventional chemotherapeutics docetaxel (DTX) and mitomycin C (MMC), which have different molecular structures and mechanisms of action than platinum-based chemotherapeutics. DU-145 PCa cells were treated with DTX and MMC alone or in combination with the carbon nanomaterials. The impact of the monotreatments and the combinatory treatments on cellular function was then systematically analyzed by using different experimental approaches (viability, short-term and long-term proliferation, cell death rate). DTX and MMC alone reduced the viability of PCa cells to 94% and 68%, respectively, whereas a combined treatment with CNFs led to less than 30% remaining viable cells. Up to 17- and 7-fold higher DTX and MMC concentrations were needed in order to evoke a similar inhibition of viability as mediated by the combinatory treatments. In contrast, the dose of platinum-based chemotherapeutics could only be reduced by up to 3-fold by combination with carbon nanomaterials. Furthermore, combinatory treatments with CNFs led mostly to an additive inhibition of short- and long-term proliferation compared to the individual treatments. Also, higher cell death rates were observed in combinatory treatments than in monotreatments, e.g., a combination of MMC and CNFs more than doubled the cell death rate mediated by apoptosis. Combinations with CNTs showed a similar, but less pronounced impact on cellular functions. In summary, carbon nanomaterials in combination with DTX and MMC evoked additive to partly synergistic anti-tumor effects. CNFs and CNTs possess the ability to sensitize cancer cells to a wide range of structurally diverse chemotherapeutics and thus represent an interesting option for the development of multimodal cancer therapies. Co-administration of chemotherapeutics with carbon nanomaterials could result in a reduction of the chemotherapeutic dosage and thus limit systemic side effects.

## Introduction

According to the global cancer statistics, prostate cancer (PCa) is the second most often diagnosed cancer in males worldwide and it ranks in fifth place among cancer-related deaths [1]. Localized PCa is usually treated by surgical removal of the prostate (radical prostatectomy) or by radiation therapy. Both treatment options are frequently associated with severe side effects such as incontinence and erectile dysfunction as well as gastrointestinal and genitourinary toxicity in case of radiation that ultimately result in a diminished quality of life [2]. Furthermore, up to 50% of patients with localized high-risk PCa will develop progressive disease following definite treatment and thus will require additional therapy [2–3]. Based on the efficacy and proven survival benefit of palliative chemotherapy in advanced PCa a curative treatment of localized PCa through pre-operative (neoadjuvant) chemotherapy has been discussed [3–4].

Neoadjuvant chemotherapy is routinely used in the treatment of other solid tumors such as bladder, breast and colon cancer [3–4]. To date it is not recommended for localized PCa by the current guidelines [2]. However, several clinical trials have demonstrated the efficacy and safety of a neoadjuvant systemic chemotherapy with docetaxel (DTX) in combination with hormonal therapy prior to radical prostatectomy in patients with high-risk PCa [5–7]. The advantages of a neoadjuvant chemotherapy lie in the down-staging of the malignancy leading to better tumor resectability and improved overall survival as well as earlier treatment of micrometastases [3–4]. Potential disadvantages include delay of surgery, over-treatment of low-risk patients and systemic side effects from chemotherapy [3]. For instance, the systemic application of DTX leads to severe dose-limiting toxicities manifested as neutropenia, febrile neutropenia, anemia, thrombocytopenia, sensory neuropathy, nausea, diarrhea, fluid retention, nail changes, and excessive tearing [8]. In order to minimize systemic side effects, a local and precise application of chemotherapeutics to the PCa foci would be an alternative approach.

In the last decades, various nanoparticles such as carbon nanotubes (CNTs) and carbon nanofibers (CNFs) have been extensively investigated for their utilization as drug carriers and delivery vehicles. They possess great potential for such biomedical applications based on their ability to be loaded with various therapeutic drugs and to be internalized by cells. Furthermore, the functionalization with targeting ligands against cancer cell-specific molecules such as antibodies, peptides or aptamers represents another advantage of carbon nanomaterials [9–12]. We and others have previously shown that CNTs can be charged with chemotherapeutics, which can eventually be released from the CNT carrier and thus exert anti-proliferative effects on cancer cells in vitro and in vivo [13–22].

Carbon nanomaterials can also augment the cell-damaging effects of conventional chemotherapeutics and cytotoxic agents by chemosensitizing cancer cells [16,23–26]. In our previous studies, CNTs and CNFs sensitized prostate and bladder cancer cells to the platinum-based chemotherapeutics carboplatin (CP) and cisplatin (CDDP) via an enhanced inhibition of short- and long-term proliferation as well as by an increased induction of apoptosis [27–28]. In order to further verify this concept and to avoid a bias that might be caused by the structurally similar platinum-based chemotherapeutics, we investigated other chemotherapeutics relevant for urological cancers, namely DTX and mitomycin C (MMC), regarding their cytotoxic effects when applied in combination with carbon nanomaterials. DTX is a cytostatic taxane approved for the palliative treatment of castration-resistant PCa [29]. It promotes microtubule stabilization, acts anti-mitotic and initiates apoptosis resulting in cell death. MMC is a cytotoxic antibiotic commonly used for the instillation treatment of non-muscle-invasive bladder cancer [30] as well as for intraperitoneal lavage of peritoneal carcinomatosis from appendiceal, colorectal and gastric cancers [31]. Following enzymatic activation MMC eventually induces a cell-cycle arrest and apoptosis via DNA crosslinking. The present study investigated the influence of CNFs and CNTs co-exposed with DTX and MMC on cellular function of PCa cells in comparison to the individual effects.

## Results

### Effect on cellular viability

**Figure 1:**
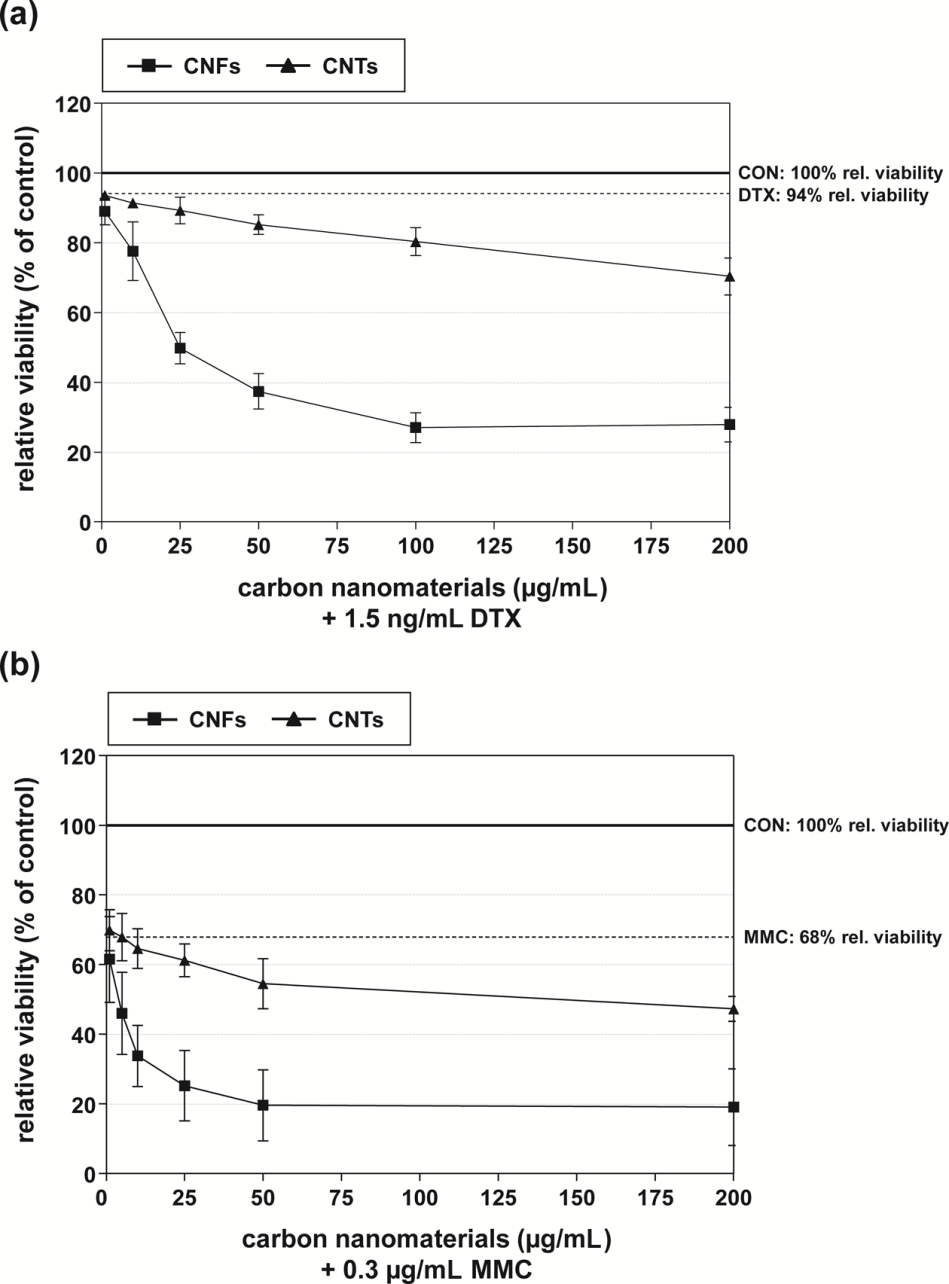
Relative cellular viability of DU-145 cells treated with (a) 1.5 ng/mL DTX or (b) 0.3 µg/mL MMC in combination with increasing concentrations of CNFs and CNTs (1–200 µg/mL), respectively. Results are depicted as averaged relative cellular viability (%) ± relative mean deviation. Untreated cells (CON) served as control (100%). The cellular viability rate following exposure to the respective chemotherapeutic alone is also indicated.

First, the influence of carbon nanomaterials and chemotherapeutics alone and in combination on cellular viability was analyzed in DU-145 PCa cells by using the WST-1 assay. In combination with at least two or more independent test systems the WST-1 assay can be recommended for evaluating the cellular effects of carbon nanomaterials, because no interference between the water-soluble formazan dye and CNTs has been detected [32]. As reported previously, both CNFs and CNTs impaired cellular viability only marginally in addition to low to moderate effects on cellular proliferation and clonogenic survival [28]. Compared to untreated control cells, both carbon nanomaterials (1–200 µg/mL) exhibited a significant inhibition of cellular viability only in concentrations above 25 µg/mL with CNFs being more detrimental (Supporting Information File 1, Table S1). In order to detect any potential synergistic effects in the combinatory treatments, DTX (1.5 ng/mL) and MMC (0.3 µg/mL) were applied in rather low concentrations. At these concentrations, DTX and MMC alone impaired the viability of DU-145 cells by about 6% and 32%, respectively (Figure 1 and Supporting Information File 1, Table S1). Both CNFs and CNTs markedly enhanced the inhibitory effect of the chemotherapeutics in a concentration-dependent manner when administered in combination (Figure 1 and Supporting Information File 1, Table S1). In detail, CNFs (≥10 µg/mL) in combination with DTX or MMC evoked significantly diminished viabilities in comparison to the individual treatments (Supporting Information File 1, Table S1). Compared to the individual effect of the chemotherapeutics, 50 µg/mL CNFs produced an additional decrease of cellular viability by about 55% and 50% in combination with DTX or MMC, respectively (Table 1, Figure 1a, and Supporting Information File 1, Table S1). This corresponded to a 2.3- and 1.5-fold synergistic increase, respectively, of the expected additive inhibition calculated from the individual treatments (Table 1). In order to achieve similar inhibition rates of about 60% and 80% mediated by 1.5 ng/mL DTX and 0.3 µg/mL MMC, respectively, in combination with 50 µg/mL CNFs (Table 1), DTX or MMC alone had to be applied in concentrations of about 25 ng/mL and 2 µg/mL, respectively (Supporting Information File 1, Figure S1a,b). This corresponded to a reduction of the chemotherapeutic dosage down to a seventeenth and a seventh, respectively, in the combinatory treatments in comparison to the individual chemotherapeutic treatment (Table 2). The combination of either chemotherapeutic with CNTs led to less pronounced effects on cellular viability, which were mostly of an additive nature (Table 1, Figure 1b, Supporting Information File 1, Table S1). In this case, DTX and MMC concentrations of about 5 ng/mL and 0.75 µg/mL were needed (Supporting Information File 1, Figure S1a,b) to evoke comparable inhibition rates of 15% and 45% of 1.5 ng/mL DTX and 0.3 µg/mL MMC, respectively, in combination with 50 µg/mL CNTs (Table 1). In both cases, this corresponded to a reduction down to approximately a third of the chemotherapeutic concentration when carbon nanomaterials were used in combination (Table 2).

**Table 1:** Effect on (A) cellular viability, (B) cellular proliferation, (C) cell colony formation and (D) cell death rate of DU-145 cells following treatment with carbon nanomaterials and chemotherapeutics alone or in combination.^a^

	CNFs		CNTs	
	DTX	MMC	DTX	MMC

(A) inhibition rate of cellular viability (%)				

chemotherapeutic alone (DTX: 1.5 ng/mL, MMC: 0.3 µg/mL)	5.9 ± 3.5	32.1 ± 9.2^b^	5.9 ± 3.5	32.1 ± 9.2^b^
carbon nanomaterial alone (50 µg/mL)	21.3 ± 2.4^b^		9.9 ± 3.9^b^	
combination: expected effect^c^	27.2	53.4	15.8	42.0
combination: measured effect^d^	62.6 ± 5.1^b,e,f^	80.4 ± 10.2^b,e,f^	14.8 ± 2.8^b^	45.5 ± 7.2^b,e^
***n*-fold increase of expected effect^g^**	**2.3**	**1.5**	**0.9**	**1.1**

(B) Inhibition rate of cellular proliferation (%)				

chemotherapeutic alone (DTX: 2.5 ng/mL, MMC: 0.5 µg/mL)	30.5 ± 1.5^b^	71.0 ± 0.7^b^	30.5 ± 1.5^b^	71.0 ± 0.7^b^
carbon nanomaterial alone (50 µg/mL)	43.0 ± 1.3^b^		18.7 ± 1.8^b^	
combination: expected effect^c^	73.5	114.0	49.2	89.7
combination: measured effect^d^	70.0 ± 5.0^b,e,f^	89.7 ± 2.4^b,e,f^	42.5 ± 3.5^b,e,f^	76.3 ± 4.9^b,e^
***n*-fold increase of expected effect^g^**	**1.0**	**0.8**	**0.9**	**0.9**

(C) inhibition rate of cell colony formation (%)				

chemotherapeutic alone (DTX: 2.5 ng/mL, MMC: 0.5 µg/mL)	28.0 ± 8.5^b^	75.5 ± 8.8^b^	28.0 ± 8.5^b^	75.5 ± 8.8^b^
carbon nanomaterial alone (50 µg/mL)	21.1 ± 9.1		17.6 ± 8.2	
combination: expected effect^c^	49.1	96.6	45.6	93.1
combination: measured effect^d^	65.3 ± 1.3^b,e,f^	88.3 ± 3.8^b,e^	43.1 ± 3.2^b,e^	73.8 ± 8.4^b,e^
***n*-fold increase of expected effect^g^**	**1.3**	**0.9**	**0.9**	**0.8**

(D) cell death rate (apoptosis & necrosis) (%)				

chemotherapeutic alone (DTX: 2.5 ng/mL, MMC: 0.5 µg/mL)	10.5 ± 0.1	17.3 ± 1.9^b^	10.5 ± 0.1	17.3 ± 1.9^b^
carbon nanomaterial alone (50 µg/mL)	12.0 ± 0.7		6.3 ± 0.9	
combination: expected effect^c^	22.4	29.3	15.8	23.6
combination: measured effect^d^	19.0 ± 2.7^b,e,f^	40.0 ± 3.1^b,e,f^	9.5 ± 0.1	21.5 ± 2.6^b,e^
***n*-fold increase of expected effect^g^**	**0.8**	**1.4**	**0.6**	**0.9**

^a^DU-145 cells were treated with DTX and MMC (concentrations as indicated) or carbon nanomaterials (50 µg/mL) alone as well as with their combinations. For cellular viability, proliferation and cell colony formation results are indicated as averaged relative inhibition (%) ± relative mean deviation; untreated cells served as control. For cell death rate averaged fractions of dead cells (%) due to apoptosis and necrosis ± mean deviation are depicted. ^b^*p* < 0.05 treatment versus control; ^c^Expected effect is the additive result calculated from the single treatments. ^d^Measured effect is the actual result following treatment with a combination of carbon nanomaterial and chemotherapeutic. ^e^*p* < 0.05 treatment versus carbon nanomaterial alone; ^f^*p* < 0.05 treatment versus chemotherapeutic alone; ^g^*n*-Fold increase of expected effect is calculated as the ratio of measured effect to expected effect.

**Table 2:** Viability inhibition rates of the combinatory treatments in comparison to the monotreatments with chemotherapeutics based on present and previous findings [28].^a^

	DTX	MMC	CDDP	CP

chemotherapeutic concentration used in combination treatments	1.5 ng/mL	0.3 µg/mL	0.25 µg/mL	7.5 µg/mL
inhibition rate in combination with CNFs (50 µg/mL)	63%	80%	60%	65%
chemotherapeutic concentration needed in monotreatment to achieve similar inhibition rate^b^	25 ng/mL	2 µg/mL	0.75 µg/mL	11 µg/mL
***n*-fold increase of chemotherapeutic dose** (monotreatment vs combination with CNFs)^c^	**16.7**	**6.7**	**3.0**	**1.5**
inhibition rate in combination with CNTs (50 µg/mL)	15%	45%	36%	43%
chemotherapeutic concentration needed in monotreatment to achieve similar inhibition rate^b^	5 ng/mL	0.75 µg/mL	0.5 µg/mL	7.5 µg/mL
***n*-fold increase of chemotherapeutic dose** (monotreatment vs combination with CNTs)^c^	**3.3**	**2.5**	**2.0**	**1.0**

^a^DU-145 cells were treated with DTX, MMC, CDDP or CP alone or in combination with carbon nanomaterials (50 µg/mL). For DTX and MCC, the cells were treated for 22 h with the carbon nanomaterials and then the chemotherapeutics were added for another 2 h. For CDDP and CP, the cells were treated simultaneously with the chemotherapeutics and the carbon nanomaterials for 24 h. Monotreatment with chemotherapeutics was 2 h for DTX and MMC or 24 h for CDDP and CP. Assessment of cellular viability was conducted 72 h after end of treatment. For further details please see the Experimental section and the previous study [28]. ^b^For dose-response curves of chemotherapeutics see Supporting Information File 1: Figure S1. ^c^*n*-Fold increase of chemotherapeutic dose is calculated as the ratio of chemotherapeutic concentration in monotreatment to chemotherapeutic concentration in combinatory treatment.

### Effect on short- and long-term cellular proliferation

**Figure 2:**
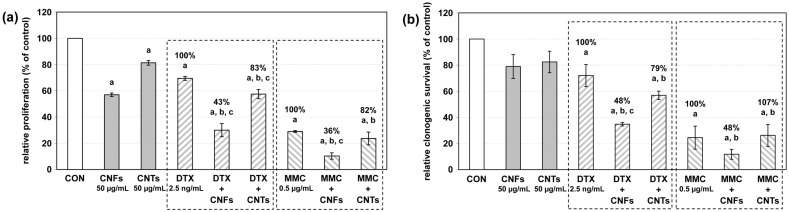
(a) Cellular proliferation and (b) clonogenic survival rate of DU-145 cells following treatment with carbon nanomaterials and chemotherapeutics alone or in combination. Combinatory treatments contained the respective carbon nanomaterial and chemotherapeutic in the same concentrations as indicated for the individual treatments. Results are depicted as averaged relative cellular proliferation or clonogenic survival (%) ± relative mean deviation. Untreated cells (CON) served as control (100%). ^a^*p* < 0.05 treatment versus control; ^b^*p* < 0.05 treatment versus carbon nanomaterial alone; ^c^*p* < 0.05 treatment versus chemotherapeutic alone.

Next, the influence of the single and combinatory treatments on cell growth was evaluated. Similar to the previous study [27], CNFs and CNTs alone (50 µg/mL) significantly inhibited cellular short-term proliferation by about 40% and 20%, respectively (Figure 2a). In contrast, both carbon nanomaterials non-significantly suppressed the clonogenic survival, which is a measure for long-term proliferation, only by about 20% (Figure 2b). As expected, DTX (2.5 ng/mL) and MMC (0.5 µg/mL) alone promoted a significant inhibition by about 30% and 75%, respectively, of both short- and long-term proliferation (Figure 2). This inhibitory effect on cell growth was further enhanced when the chemotherapeutics were administered in combination with CNFs or CNTs (50 µg/mL). Combinations with CNFs significantly decreased short-term proliferation down to 43% and 36%, respectively, compared to the inhibition mediated by DTX and MMC alone when set to 100% (Figure 2a). CNTs produced an additional inhibition of cellular proliferation by about 20% compared to either chemotherapeutic alone, which was only significant for the combination with DTX (Figure 2a). Compared to DTX and MMC alone (set to 100%), CNFs also diminished long-term proliferation by about 50% in combination with either chemotherapeutic, albeit this was only significant for the combination with DTX (Figure 2b). In contrast, CNTs led only to a noticeable reduction of clonogenic survival of about 20% in combination with DTX (Figure 2b). In summary, the inhibitory effects of the combinatory treatments on short- and long-term proliferation were mostly of an additive to partly synergistic nature (Table 1).

### Effect on cell death rate

**Figure 3:**
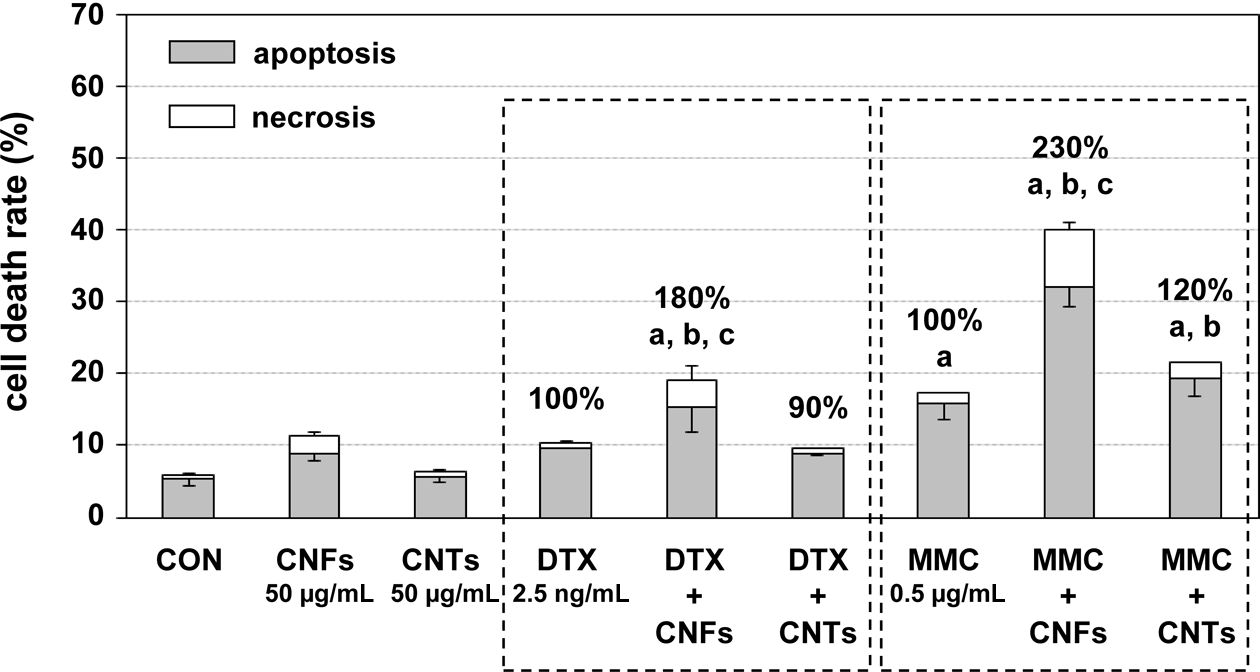
Cell death rate of DU-145 cells following treatment with carbon nanomaterials and chemotherapeutics alone or in combination. Combinatory treatments contained the respective carbon nanomaterial and chemotherapeutic in the same concentrations as indicated for the individual treatments. Averaged fractions of dead cells (%) due to apoptosis and necrosis ± mean deviation are depicted. Untreated cells (CON) served as control treatment. ^a^*p* < 0.05 treatment versus control; ^b^*p* < 0.05 treatment versus carbon nanomaterial alone, ^c^*p* < 0.05 treatment versus chemotherapeutic alone.

**Figure 4:**
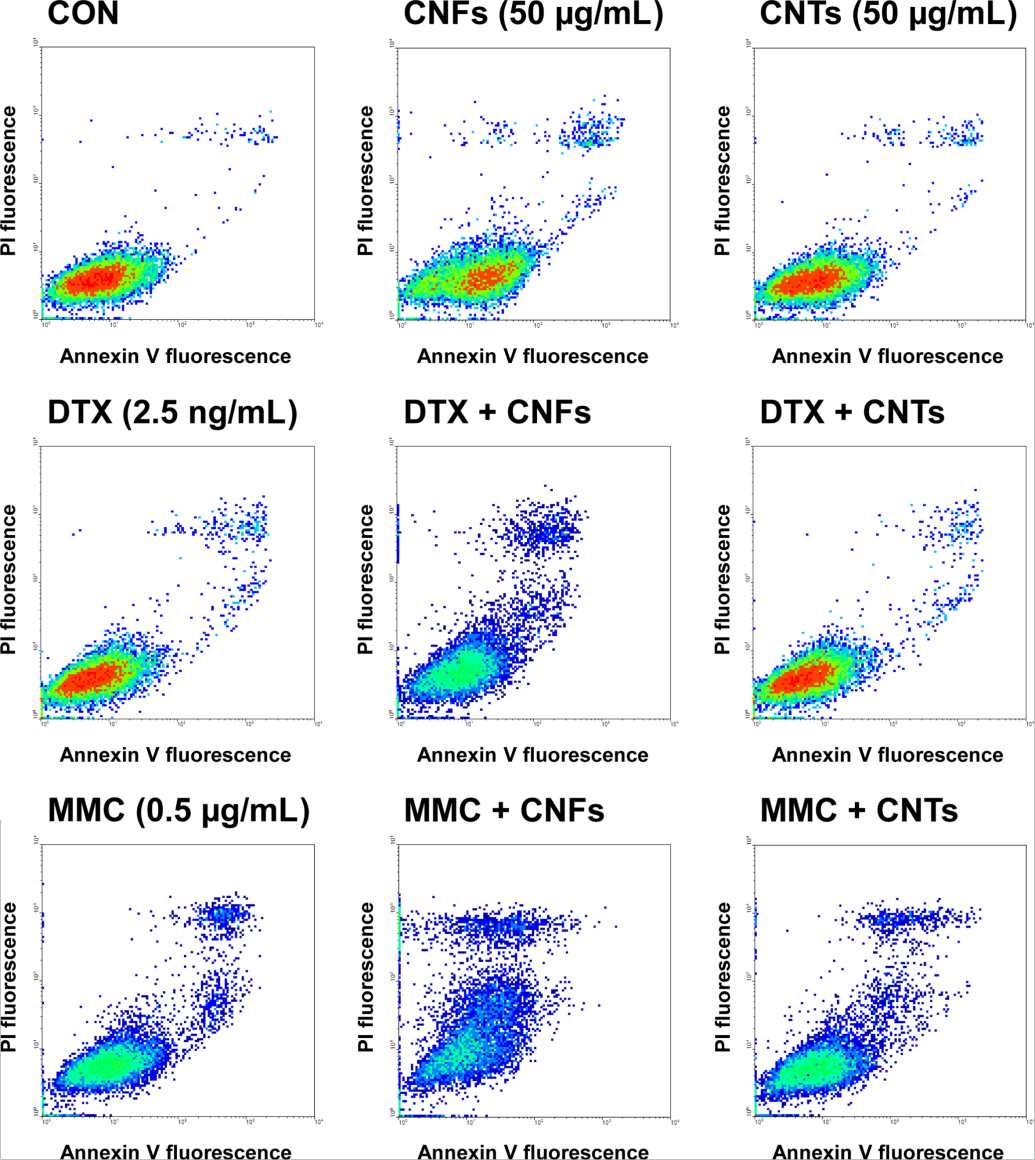
Exemplary flow cytometry analysis for cell death of DU-145 cells following treatment with carbon nanomaterials and chemotherapeutics alone or in combination. Combinatory treatments contained the respective carbon nanomaterial and chemotherapeutic in the same concentrations as indicated for the individual treatments. Untreated cells (CON) served as control treatment.

Both chemotherapeutics mediate cell death via the initiation of apoptosis. Consequently, the effect of the single and combinatory treatments on apoptosis- and necrosis-mediated cell death was determined. A treatment with carbon nanomaterials or chemotherapeutics alone elevated the cell death rates only marginally to moderately (Figure 3 and Figure 4). Co-exposure of DU-145 cells with CNFs and DTX or MMC led to significantly increased cell death rates by additional 80% and 130%, respectively (Figure 3). Notably, apoptosis and not necrosis mainly contributed to the enhanced cell death rate (Figure 3 and Figure 4). In contrast, CNTs produced an increase in cell death rate by about 20% only in combination with MMC, which was, however, not significant compared to the effect mediated by MMC alone (Figure 3). Overall, the combinatory treatments led to a mostly additive to partly synergistic induction of the cell death rate (Table 1).

## Discussion

In addition to surgery and radiotherapy chemotherapy is the third pillar in cancer treatment. In PCa, systemic chemotherapy with DTX is mainly applied for the treatment of advanced PCa stages, whereas neoadjuvant chemotherapy prior surgery has not been implemented in the management of localized PCa [2,29]. Based on the efficacy of a DTX therapy in the palliative setting several phase I/II trials have examined a neoadjuvant DTX therapy in combination with androgen deprivation in high-risk PCa demonstrating its usefulness and safety [5–7]. However, systemic chemotherapy is associated with severe side effects limiting the maximum drug dose and resulting in a diminished quality of life for the patients. Furthermore, the effective delivery and homogenous distribution of small molecules such as chemotherapeutics at the tumor site is hindered by the high interstitial fluid pressure of solid tumors [9]. In contrast, macromolecular compounds such as CNFs and CNTs can accumulate into solid tumors much easier by taking advantage of the enhanced permeability and retention effect, which is caused by the inconsistent blood flow, leaky vasculature and impaired lymphatic drainage of the tumor tissue [9–10]. Therefore, a combination of low-molecular chemotherapeutics with suitable nanocarriers could enhance the drug accumulation and retention in the tumor tissue. Furthermore, a local and precise administration of such complex drug delivery systems at the tumor site would minimize systemic resorption and deleterious effects to healthy tissues. Carbon nanomaterials such as CNFs and CNTs represent a much investigated option for such biomedical applications.

In addition to facilitate a passive targeting, various studies have demonstrated that carbon nanomaterials can also act as anti-tumor agents themselves and sensitize cancer cells to cytotoxic drugs such as etoposide [23–24,26], dexamethasone [23], paclitaxel [25] and CDDP [16]. In accordance, we have previously shown that CNFs and multiwalled CNTs could enhance the anti-proliferative and pro-apoptotic effects of CDDP and CP in prostate and bladder cancer cells [27]. In order to avoid a bias, we investigated in the present study the conventional chemotherapeutics DTX and MMC, which have different molecular structures and mechanisms of action than platinum-based chemotherapeutics. We systematically analyzed the impact of monotreatments and combinatory treatments on cellular function by employing different experimental approaches (viability, short-term and long-term proliferation, cell death rate).

In the present study, the impairment of cellular function of DU-145 PCa cells mediated by DTX and MMC could be further enhanced when applied in combination with CNFs or CNTs (50 µg/mL). Particularly, combinatory treatments with CNFs and either chemotherapeutic evoked an additive to partly synergistic inhibition of cellular viability, short- and long-term proliferation as well as induction of apoptosis. In contrast, combinations with CNTs led to less pronounced cellular effects, which were of an additive nature. These results are similar to our previous findings for combination of CNFs–CNTs (50 µg/mL) with CDDP and CP (Table 3) [27–28]. In detail, almost all investigated chemotherapeutics led to a synergistically enhanced inhibition of cellular viability in combination with carbon nanomaterials, particularly with CNFs (Table 3). The highest increase of the expected inhibition of 2.3-fold was observed for the combination of CNFs and DTX. In contrast, mostly additive effects were achieved for the inhibition of proliferation and cell colony formation as well as induction of cell death rate regardless of the chemotherapeutic (Table 3). However, synergistic increases of the expected effects of 1.3-, 1.4- and 1.6-fold could be observed for the combinations CNFs–DTX (cell colony formation), CNFs–MMC (cell death rate) and CNFs–CP (cell death rate), respectively (Table 3). Furthermore, other studies have also demonstrated that single-walled CNTs can augment the effectiveness of cytotoxic drugs such as etoposide and paclitaxel via enhanced apoptosis [24–26]. The single-walled CNTs have been applied in concentrations of up to 20 µg/mL in these studies [24–26], whereupon one has to bear in mind that single-walled CNTs are more toxic than multiwalled CNTs [33].

**Table 3:** Effects of combinatory treatments with DTX and MMC in comparison to previous findings with CDDP and CP [27–28].^a^

	DTX	MMC	CDDP	CP

*n*-fold increase of cellular viability inhibition^b^				

combinations with CNFs	2.3	1.5	1.7	1.6
combinations with CNTs	0.9	1.1	1.5	1.5

*n*-fold increase of cellular proliferation inhibition^b^				

combinations with CNFs	1.0	0.8	0.9	0.8
combinations with CNTs	0.9	0.9	0.9	0.9

*n*-fold increase of cell colony formation inhibition^b^				

combinations with CNFs	1.3	0.9	1.1	1.0
combinations with CNTs	0.9	0.8	1.0	0.9

*n*-fold increase of cell death rate (apoptosis & necrosis)^b^				

combinations with CNFs	0.8	1.4	0.9	1.6
combinations with CNTs	0.6	0.9	0.7	0.9

^a^DU-145 cells were treated with DTX, MMC, CDDP or CP in combination with carbon nanomaterials (50 µg/mL). For DTX and MCC, the cells were treated for 22 h with the carbon nanomaterials and then the chemotherapeutics were added for another 2 h. For CDDP and CP, the cells were treated simultaneously with the chemotherapeutics and the carbon nanomaterials for 24 h. Assessment of cellular function was conducted 72 h after end of treatment. For further details please see the Experimental section and the previous studies [27–28]. ^b^*n*-Fold increase of expected effect is calculated as the ratio of measured effect to expected effect.

In order to mimic similar viability inhibition rates mediated by the combinatory treatments, DTX and MMC had to be applied in concentrations up to 17- and 7-fold higher than in the combinatory treatments. In contrast, the dose of platinum-based chemotherapeutics could only be reduced down to a third by combination with carbon nanomaterials (Table 2 and Supporting Information File 1, Figure S1c,d) [28]. Consistently, the reduction of the chemotherapeutic dosage was more distinct in combinatory treatments with CNFs independent of the chemotherapeutic used. A combined application with carbon nanomaterials would also result in an improved cellular uptake of the active drug component as previously shown by some studies [26,28]. Therefore, the co-administration of chemotherapeutics with carbon nanomaterials could result in a reduction of the chemotherapeutic dosage and thus in the reduction of severe side effects as well as in the circumvention of chemoresistance.

To the best of our knowledge, this is the first study demonstrating that the cytotoxic effects of MMC can be augmented when applied in combination with carbon nanomaterials. Consistent with our findings, DTX adsorbed to single-walled CNTs led to a greater inhibition of cellular viability in PCa cells compared to DTX alone [18]. In other types of cancer cells, DTX conjugated to or loaded into multiwalled CNTs, which is different from our approach, surpassed the anti-proliferative and pro-apoptotic activity of free DTX [19–22]. In contrast to our systematic evaluation, most of these studies only evaluated the rate of viability and apoptosis following treatment. An enhanced inhibition of tumor cell growth mediated by DTX–CNT complexes could also be observed in in vivo animal models for various tumor entities [18,22]. These studies suggested that DTX–CNT complexes possess a prolonged half-life and higher accumulation rate in tumor tissue compared to free DTX, which ultimately results in an improved therapeutic effect [18,22]. In addition, non-CNT nanoparticles have been functionalized with DTX and could mediate PCa growth inhibition in vitro and in vivo [34–39]. For instance, DTX encapsulated in PLGA-PEG nanoparticles evoked an improved inhibition of cancer cell and tumor tissue growth compared to free DTX [38].

The superiority of CNFs in combination treatments compared to CNTs might be explained by their different morphology (Table 4). While CNTs consist of concentric graphene cylinders with an inert surface, the herringbone-like CNFs possess exposed graphene edge planes resulting in a higher proportion of reactive defects on their surface. The presence of structural defects has been shown to contribute to the detrimental effects of carbon nanomaterials on cellular function [40]. Furthermore, CNFs were much easier internalized by DU-145 cells than CNTs and thus might better interact with cell components leading to enhanced cellular impairment [27–28]. Nevertheless, both carbon nanomaterials have been shown to adhere to the surface of DU-145 cells [28]. Due to their interaction with cancer cells, both carbon nanomaterials could act like nanoneedles and diminish the integrity of the cellular membrane leading to an enhanced intracellular drug accumulation. Accordingly, we and others have shown that the intracellular CP and etoposide content was elevated when co-administered with CNFs and single-walled CNTs, respectively [26,28]. This elevation in the intracellular drug concentration also contributes to the circumvention of chemoresistance, which is major obstacle of chemotherapy in addition to systemic side effects and can be caused by decreased drug uptake, increased drug efflux and inhibition of apoptosis [41]. Consequently, an enhanced apoptosis could also contribute to the chemosensitizing activity of carbon nanomaterials, which was demonstrated in our present and previous investigations [27]. Consistently, carbon nanomaterials have also been shown to sensitize chemoresistant cells to conventional chemotherapeutics and thus make them more prone to the cytotoxic drug effects [17,28].

**Table 4:** Physico-chemical properties of the tested carbon nanomaterials as determined previously [13,28].

	CNFs	CNTs

morphology	stacked-cup structure (herringbone-like)	20–30 parallel sidewalls
outer diameter (nm)	30–170	10–70
length (µm)	0.8–50	0.8–15
length-to-diameter aspect ratio	≈6–1700	≈14–1500

One limiting factor for the application of carbon nanomaterials as drug delivery systems are their toxicity owing to their small size and structural resemblance to asbestos [33,42]. In the present study the concentration of CNFs and CNTs in the combinatory treatments was rather high (50 µg/mL), but well within the range of in vitro analyses. However, due to the aforementioned enhanced permeability and retention effect of solid tumor tissue, the concentration of carbon nanomaterials can probably be further diminished for in vivo testing. Furthermore, the toxicity of carbon nanomaterials might depend on the route of administration with systemically applied nanocarriers being the most toxic [33,42]. In in vivo animal studies the adverse effects of systemically applied CNTs resulted from their accumulation in major organs, such as lung, liver and spleen [33]. In contrast, CNTs precisely delivered to subcutaneous tissue induced only short-term immunological reactions, showed no evidence of local or systemic toxicity and only a small amount of CNTs entered the bloodstream [43–44]. Consequently, a local application of nanomaterial-based drug delivery systems directly to the tumor site would limit their systemic resorption and thus adverse effects to other organs.

## Conclusion

In this study we could demonstrate that the anti-proliferative and pro-apoptotic effects of two structurally diverse chemotherapeutics, DTX and MMC, could be enhanced when administered in combination with CNFs and CNTs. Combinatory treatments with CNFs evoked additive to partly synergistic anti-tumor effects, whereas combinations with CNTs led to less pronounced, but additive cellular effects. Together with our previous findings, this study shows that CNFs and CNTs can enhance the cytotoxic effects of a wide range of structurally diverse chemotherapeutics. Thus, the development of multimodal therapies based on carbon nanomaterials seems very promising, as carbon nanomaterials possess great potential for functionalization and combination with other therapy approaches. CNFs or CNTs as drug delivery systems would also permit a more precise and minimal invasive application to the tumor site such as PCa foci. Ultimately, the local co-administration of chemotherapeutics with carbon nanomaterials could result in a reduction of the chemotherapeutic dosage and thus limit systemic side effects and prevent chemoresistance.

## Experimental

### Carbon nanomaterials and chemotherapeutics

Two types of carbon nanomaterials were used: multiwalled CNTs [45] and CNFs (PR-24-XT-HHT; Pyrograf Products Inc., Cedarville, OH, USA). The physico-chemical properties of both carbon nanomaterials have been determined previously [13,28] and are summarized in Table 4. Chemotherapeutics were provided by the in-house pharmacy (University Hospital, Dresden, Germany). Dispersions of the carbon nanomaterials were freshly prepared as previously described [27–28].

### Cell culture

The human PCa cell line DU-145 (HTB-81; ATCC, Rockville, MD, USA) was cultured in Dulbecco’s modified minimum essential medium supplemented with 10% fetal bovine serum, 1% 1 M HEPES buffer and 1% MEM non-essential amino acids (all from Invitrogen, Karlsruhe, Germany) at standard conditions (37 °C, humidified atmosphere containing 5% CO_2_). During treatment 1% streptomycin/penicillin (Invitrogen) was added to the cell culture medium.

Cells were cultured in microplates and subsequently treated with the indicated agents. Individual treatments with carbon nanomaterials (1–200 µg/mL) or chemotherapeutics (DTX: 1.5–2.5 ng/mL, MMC: 0.3–0.5 µg/mL) were conducted for 24 h or 2 h, respectively. For combinatory treatments the respective solutions with the chemotherapeutic or the carbon nanomaterial were prepared separately. Then, the cells were first treated with carbon nanomaterials alone for 22 h, afterwards the respective chemotherapeutic was added for another 2 h. Following incubation, the cells were washed twice with PBS and incubated with fresh cell culture medium for another 72 h followed by the conduction of the cellular assays.

### Cellular viability assay

Cellular viability was quantified in quintuplicate in 96-well culture plates using the cell proliferation reagent WST-1 (Roche, Mannheim, Germany), for which no interaction between the water-soluble formazan dye and CNTs has been observed [32]. WST-1 reagent was added to the cells 72 h after end of treatment according to the instructions of the manufacturer. Absorbance was measured with a spectrophotometer (Anthos labtec, Krefeld, Germany) at 450 nm with 620 nm as reference wavelength and was directly proportional to the viability of the cells.

### Cell proliferation, clonogenic survival and cell death rate

For examination of cell proliferation, cell colony formation and apoptosis cells were seeded into 6-well plates and incubated as indicated above. Seventy two hours after the end of treatment cells were harvested by trypsin/EDTA treatment and submitted to the aforementioned assays. Cell proliferation was measured by counting the number of cells present in the samples using a Z2 Coulter counter (Beckman Coulter, Brea, CA, USA). Subsequently, 200 pretreated cells per well were seeded in triplicates into 6-well plates to observe their ability to form colonies (clonogenic assay). Following incubation for 8 to 11 days, colonies were fixed with 4% buffered formaldehyde, washed with PBS and stained with Giemsa solution (diluted with water 1:20; Merck, Darmstadt, Germany). Macroscopically visible colonies were then counted. For evaluation of the apoptosis rate, the Annexin V FITC Apoptosis Detection Kit I (BD Biosciences, Heidelberg, Germany) was used. The cells were prepared according to the recommendation of the provider followed by the analysis of 20,000 cells by flow cytometry (FACScan, BD Biosciences). Using the quadrant analysis mode of WinMDI 2.8 software (The Scripps Research Institute, La Jolla, CA, USA) cells were classified as non-apoptotic (not stained), early apoptotic (positive for Annexin V only), late apoptotic (positive for Annexin V and propidium iodide) or necrotic (positive for propidium iodide only).

### Statistics

For each treatment two to six independent experiments were performed. For cellular viability, cellular proliferation and cell colony formation the averaged results were expressed as percentage of control ± relative mean deviation; the control values were set 100%. For cell death rate averaged fractions of dead cells (%) due to apoptosis and necrosis ± mean deviation were reported. Statistical differences were evaluated using analysis of variance (ANOVA) followed by Bonferroni’s correction for multiple comparisons. A p-value smaller than 0.05 was considered statistically significant. All statistical calculations were performed using GraphPad Prism 5.03 (GraphPad Software, San Diego, CA, USA).

## Supporting Information

Additional experimental data.

File 1 Relative cellular viability of DU-145 cells following monotreatment or combinatory treatment including dose-response curves.
