# Supplementary material for: Carbon nanomaterials sensitize prostate cancer cells to docetaxel and mitomycin C via induction of apoptosis and inhibition of proliferation
Source: Beilstein J Nanotechnol. 2017 Jun 23;8:1307–17. doi: 10.3762/bjnano.8.132 (PMC5496539; doi:10.3762/bjnano.8.132)
Supplement: File 1 — Relative cellular viability of DU-145 cells following monotreatment or combinatory treatment including dose-response curves. [file Beilstein_J_Nanotechnol-08-1307-s001.pdf]

Supporting Information

for

Carbon nanomaterials sensitize prostate cancer cells to docetaxel and mitomycin C via induction of apoptosis and inhibition of proliferation

Kati Erdmann*[‡,1], Jessica Ringel[‡,1], Silke Hampel[2], Manfred P. Wirth[1] and Susanne Fuessel[1]

Address: [1]Department of Urology, Medizinische Fakultät Carl Gustav Carus, Technische Universität Dresden, Fetscherstraße 74, Dresden 01307, Germany and [2]Leibniz Institute of Solid State and Material Research Dresden, P.O. Box 270016, Dresden 01171, Germany

Email: Kati Erdmann* - kati.erdmann@uniklinikum-dresden.de

* Corresponding author

[‡]Equal contributors

Relative cellular viability of DU-145 cells following monotreatment or combinatory treatment including dose-response curves

Table S1: Relative cellular viability of DU-145 cells following treatment with carbon nanomaterials and chemotherapeutics alone or in combination.[a]

carbon nanomaterial	chemo-therapeutic	concentration carbon nanomaterial (µg/mL)							
		**0**	**1**	**5**	**10**	**25**	**50**	**100**	**200**
CNFs	none	100 (CON)	101.1 ± 2.5	97.0 ± 3.0	95.2 ± 4.8	86.9 ± 2.4[b]	78.7 ± 2.4[b]	78.3 ± 3.5[b]	79.4 ± 3.3[b]
	DTX (1.5 ng/mL)	94.1 ± 3.5	89.0 ± 3.8[b,c]	n.d.	77.6 ± 8.4[b,c,d]	49.8 ± 4.5[b,c,d]	37.4 ± 5.1[b,c,d]	27.0 ± 4.3[b,c,d]	27.9 ± 5.0[b,c,d]
	MMC (0.3 µg/mL)	67.9 ± 9.2[b]	61.5 ± 12.3[b,c]	46.0 ± 11.8[b,c,d]	33.8 ± 8.8[b,c,d]	25.2 ± 10.1[b,c,d]	19.6 ± 10.2[b,c,d]	n.d.	19.1 ± 11.0[b,c,d]
CNTs	none	100 (CON)	95.7 ± 3.3	95.4 ± 3.0	95.1 ± 0.4	93.7 ± 5.3	90.1 ± 3.9[b]	88.2 ± 4.3[b]	79.6 ± 4.1[b]
	DTX (1.5 ng/mL)	94.1 ± 3.5	93.6 ± 1.0	n.d.	91.4 ± 0.4	89.3 ± 3.8	85.2 ± 2.8[b]	80.3 ± 4.0[b,d]	70.4 ± 5.3[b,d]
	MMC (0.3 µg/mL)	67.9 ± 9.2[b]	69.9 ± 5.9[b,c]	67.9 ± 6.8[b,c]	64.6 ± 5.7[b,c]	61.2 ± 4.7[b,c]	54.5 ± 7.2[b,c]	n.d.	47.3 ± 3.6[b,c,d]

[a]Combinatory treatments contained the respective carbon nanomaterial and chemotherapeutic in the same concentrations as indicated for the individual treatments. Results are depicted as averaged relative cellular viability (%) ± relative mean deviation. Untreated cells (CON) served as control (100%). The effects of CNFs and CNTs on cellular viability have been reported previously [28]; n.d.: not determined.

[b]$p < 0.05$ treatment versus control, [c]$p < 0.05$ treatment versus carbon nanomaterial alone, [d]$p < 0.05$ treatment versus chemotherapeutic alone

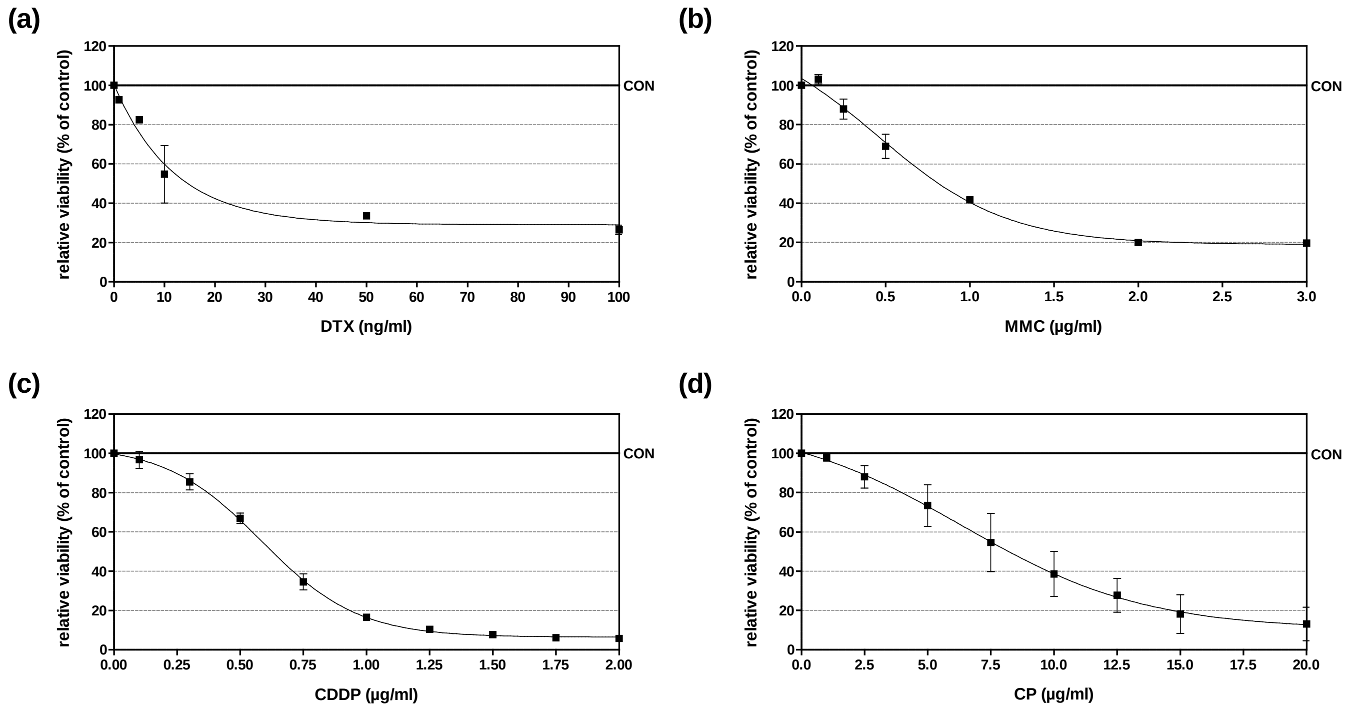

Figure S1: Relative cellular viability of DU-145 cells treated with increasing concentrations of (a) DTX, (b) MMC, (c) CDDP and (d) CP. Results are depicted as averaged relative cellular viability (%) ± relative mean deviation. The effects of CDDP and CP on cellular viability have been reported previously [28]. Cells were treated for 2 h (DTX, MMC) or 24 h (CDDP, CP) with increasing concentrations of the chemotherapeutics. Assessment of cellular viability was conducted 72 h after end of treatment. Untreated cells (CON) served as control (100%). The 50% inhibitory concentrations (IC50) calculated from the dose-response curves were 8.96 ng/mL for DTX, 0.63 µg/mL for MMC, 0.62 µg/mL for CDDP and 8.35 µg/mL for CP.
